# Learning from the value of your mistakes: evidence for a risk-sensitive process in movement adaptation

**DOI:** 10.3389/fncom.2013.00118

**Published:** 2013-08-23

**Authors:** Michael C. Trent, Alaa A. Ahmed

**Affiliations:** Neuromechanics Laboratory, Department of Integrative Physiology, University of Colorado, BoulderBoulder, CO, USA

**Keywords:** risk-sensitivity, adaptation, motor learning, decision-making, internal model, subjective value, sensorimotor control

## Abstract

Risk frames nearly every decision we make. Yet, remarkably little is known about whether risk influences how we learn new movements. Risk-sensitivity can emerge when there is a distortion between the absolute magnitude (actual value) and how much an individual values (subjective value) a given outcome. In movement, this translates to the difference between a given movement error and its consequences. Surprisingly, how movement learning can be influenced by the consequences associated with an error is not well-understood. It is traditionally assumed that all errors are created equal, i.e., that adaptation is proportional to an error experienced. However, not all movement errors of a given magnitude have the same subjective value. Here we examined whether the subjective value of error influenced how participants adapted their control from movement to movement. Seated human participants grasped the handle of a force-generating robotic arm and made horizontal reaching movements in two novel dynamic environments that penalized errors of the same magnitude differently, changing the subjective value of the errors. We expected that adaptation in response to errors of the same magnitude would differ between these environments. In the first environment, Stable, errors were not penalized. In the second environment, Unstable, rightward errors were penalized with the threat of unstable, cliff-like forces. We found that adaptation indeed differed. Specifically, in the Unstable environment, we observed reduced adaptation to leftward errors, an appropriate strategy that reduced the chance of a penalizing rightward error. These results demonstrate that adaptation is influenced by the subjective value of error, rather than solely the magnitude of error, and therefore is risk-sensitive. In other words, we may not simply learn from our mistakes, we may also learn from the value of our mistakes.

## Introduction

Effective movement relies largely on adaptation: the process of correcting control from movement to movement, which, critically, is driven by movement error (Topka et al., 1998; Smith and Shadmehr, 2005; Bastian, 2008; Rabe et al., 2009). However, investigations of adaptation have largely overlooked the fact that not all movement errors of the same magnitude necessarily have the same subjective value. Consider a game of tennis. Compare a 5 cm error in ball placement in the middle of your opponent's court with a 5 cm error at the edge that lands the ball out-of-bounds. Although the error magnitude is equivalent, the subjective value is drastically different. Clearly one would respond to each error very differently on the next shot. Most movements involve similar decisions, where the subjective value of an error must be considered. The fact that people respond differently demonstrates that the magnitude of the error and the subjective value of the error, are not always the same. This raises the question of whether adaptation may depend on the subjective value, rather than simply the magnitude, of an error.

A distortion between the magnitude (actual) and the subjective value of an error suggests a risk-sensitive decision-making process in the brain (Bernoulli, 1954; Huettel et al., 2006). Sensitivity to risk, the variance associated with an outcome, has been extensively studied in economics (Kahneman and Tversky, 1979; Smith et al., 2002), psychology, and, recently, motor control (Wu et al., 2009; Braun et al., 2011; O'Brien and Ahmed, 2013). Theoretically, risk-sensitive behavior can arise from a distortion between subjective and actual value (i.e., a non-linear value function; Glimcher, 2008). Risk-sensitivity suggests, counter to traditional computational models of decision making and learning that decisions are not based solely on maximizing expected gain over all outcomes, but are also influenced by the variance. However, research on risk-sensitivity has largely focused on single decisions, with few investigating sequences of decisions in cognitive tasks (Niv et al., 2012). This is surprising, as the process of making sequences of decisions is, at its core, the learning process. Here we investigate movement adaptation: a sequential decision-making process critical to effective movements.

Adaptation is frequently studied in humans by exposing them to novel dynamic environments (Lackner and Dizio, 1994; Shadmehr and Mussa-Ivaldi, 1994; Scheidt et al., 2001). Adaptation proportional to the magnitude of error has been observed, suggesting that error magnitude is being minimized. However, the idea of proportionality between error magnitude and adaptation has been recently challenged. Non-linear adaptation has emerged when the directional bias, relevance, statistics and magnitude of the errors are manipulated (Fine and Thoroughman, 2007; Wei and Koerding, 2009; Marko et al., 2012). These results suggest that adaptation is not solely dependent on error magnitude.

We sought to determine if we could alter adaptation patterns by manipulating the subjective value of movement errors with identical magnitude. If adaptation to errors with identical magnitude differs with the subjective value of the error, this would quantitatively demonstrate that adaptation is risk-sensitive and influenced by the subjective value of error.

## Methods

### Theoretical development

**Figure 1:**
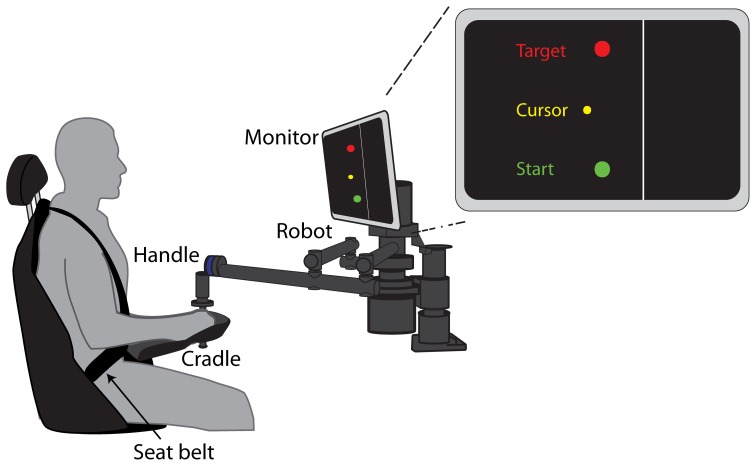
**Experimental Setup**. Seated participants grasped the handle of a robotic arm and made 15 cm reaching movements from a start circle to a target circle. The forearm was supported by a cradle attached to the robotic arm. Visual feedback of the start, target, and cursor were provided on a monitor mounted at eye level. The location of the “cliff” was indicated by the line 2.5 cm to the right of the start and target circles.

To examine the influence of the subjective value of movement error on adaptation, we created a task in which we modulated the subjective value associated with a movement error of a given magnitude. Participants made reaching movements while holding the handle of a force-generating robot arm (Figure 1) in two novel dynamic environments that imposed different consequences on the same magnitude of movement error.

The first dynamic environment, Stable, was a velocity-dependent force field, which perturbed their reaching movements and required participants to compensate in order to reach the target. The force field pushed the handle to the left, away from the target which was directly ahead. To reach the target accurately, participants had to push to the right to effectively cancel out the perturbation. The perturbation generated by the robot changed in magnitude but not direction from trial-to-trial. This is a well-studied paradigm where results have consistently demonstrated that healthy adults adapt in a manner proportional to movement error magnitude (Shadmehr and Mussa-Ivaldi, 1994; Thoroughman and Shadmehr, 2000).

**Figure 2:**
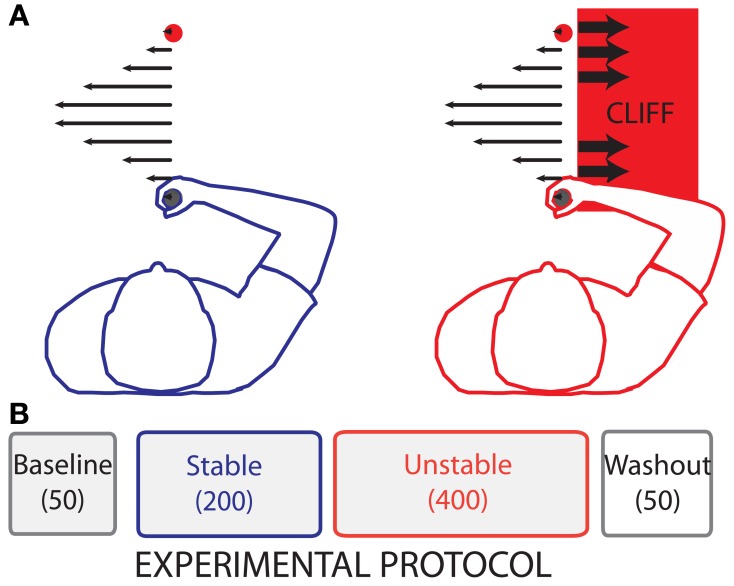
**Experimental Protocol. (A)** Illustration of the forces produced by the robot during reaching. Leftward arrows (filled) in both panels represent the viscous curl field forces. Rightward (empty) arrows in the rightmost panel represent position dependent divergent forces. **(B)** The experiment consisted of four phases: Baseline (50 no-force trials), Stable (200 trials with changing curl-field dynamics), Unstable (400 trials with changing curl-field dynamics identical to the Stable trials, but with penalties associated with large rightward movement errors), and Washout (50 no-force trials).

In the second dynamic environment, Unstable, movement errors in the right half of the screen were heavily penalized, thereby altering the subjective value of an error of a given magnitude relative to the Stable environment. The Unstable environment was identical to the Stable environment, except that we imposed a boundary on the right side of the screen, simulating a virtual cliff. Errors to the right of this boundary would lead to instability: large rightward perturbing forces that participants could not compensate for within that trial (Figure 2). On trials with such an error, it was not possible for the participants to reach the target after the cursor had crossed the boundary. Here rightward errors were considerably less desirable than leftward errors. Compared to the Stable environment, a rightward error of the same magnitude in the Unstable environment had a less desirable consequence, and therefore a different subjective value.

If adaptation was influenced by an individual's subjective value of the error, then the relationship between the magnitude of error experienced on one trial, and the adaptation observed on the following trial in response to that error would differ between movement environments. To predict the nature of this difference, we developed a risk-sensitive model of movement adaptation by building upon a commonly-used model of movement adaptation that predicts proportional adaptation to movement error (Thoroughman and Shadmehr, 2000). This model is based upon the assumption that the central nervous system seeks the motor command, *y*, which will minimize the squared movement error, *x*^2^. Solving this optimization problem, an expression is obtained for how the command on the following trial, *y*_*n* + 1_, should adapt based on the predicted perturbation on the current trial, ${\hat{B}}_{n}$, and the movement error experienced on the current trial, *x*_*n*_:

(1) $$x_{n}=D(B_{n}-{\hat{B}}_{n})$$

(2) $${\hat{B}}_{n+1}=A{\hat{B}}_{n}+Kx_{n}$$

(3) $$y_{n+1}=-{\hat{B}}_{n+1}$$

Here, *x* represents the movement error on the current trial, *n*, and results from a difference between the predicted perturbation, $\hat{B}$, and the actual perturbation, *B*, experienced on that trial. Importantly, the amount of adaptation to a given error is represented by a constant, *K*, which will lead to proportional adaptation to error. The parameters *A* and *D* represent forgetting and system compliance parameters, respectively.

In a risk-sensitive formulation, the amount of adaptation can depend on the subjective value associated with a given error: *v*(*x*), which can be a non-linear function of the errors observed. Substituting this error sensitivity function into (2), brings us to the following expression:

(4) $${\hat{B}}_{n+1}=A{\hat{B}}_{n}+Kv(x_{n})$$

While this function can theoretically take a variety of forms, we will use a simple piecewise linear function to differentially weight rightward and leftward errors, similar to functions used to describe increased sensitivity to positive vs. negative rewards (Niv et al., 2012):

(5) $$v(x)=\{\begin{array}{cc} \alpha x & \;if\;x>0,\;rightward\\ -\beta |x| & if\;x<0,\;leftward \end{array}$$

Clearly, when α = β, rightward and leftward errors are valued equally and there is no distortion between the magnitude and subjective value of an error. Effectively, we are back to (2), which can now be described as risk-neutral adaptation. However, in the Unstable phase, the addition of a cliff on the right creates a subjective error-value function that explicitly penalizes rightward errors more than leftward errors such that α > β > 0. It can now be seen how a distortion between the magnitude and subjective value of an error will manifest as risk-sensitivity in the learning process which arises from sensitivity to outcome variance. The non-linear transformation of movement errors results in asymmetric learning from rightward and leftward errors. This asymmetry will lead to outcome variance being penalized or favored.

**Figure 3:**
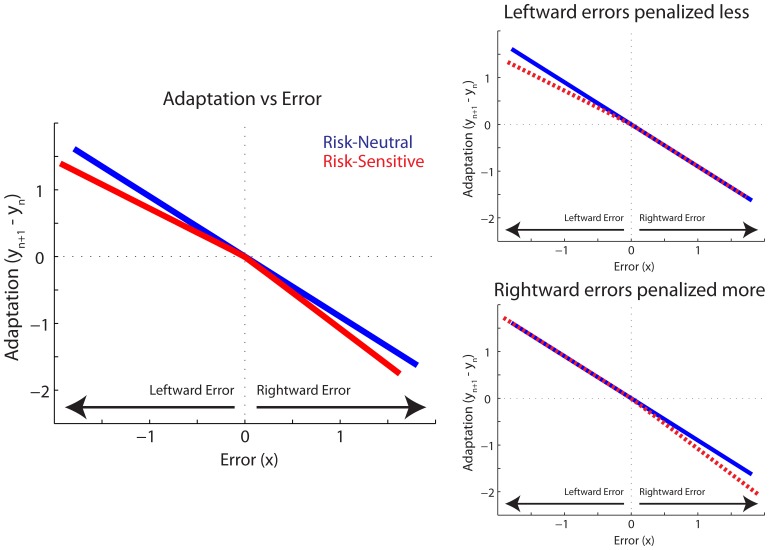
**A Risk-Sensitive Model of Movement Adaptation**. Predicted Adaptation is plotted vs. Error for Risk-Neutral (blue line) and Risk-Sensitive (red) models. Error represents positional error, *x*, on trial *n*. Adaptation represents the change in command, y, from trial *n* to *n* + 1. **Left panel** plots risk-sensitive predictions when leftward errors are penalized less and rightward errors are penalized more. Also depicted are risk-sensitive model predictions for when only leftward errors are penalized less **(top right panel**), and when only rightward errors are penalized more (**bottom right panel**). Risk-sensitive predictions plotted with dashed red lines in right panels for clarity.

The risk-sensitive model described above was used to simulate adaptation to a random sequence of perturbation gains and the results are shown in Figure 3. This sequence of perturbation gains replicated that which was experienced by the participants. Adaptation was calculated as the difference between the motor commands on two subsequent trials: *y*_*n* + 1_ − *y*_*n*_. Model parameters were set arbitrarily at *A* = 1, *K* = 0.9, and *D* = 0.1. Importantly, the qualitative predictions presented in Figure 3 are not sensitive to these particular parameter values. Rightward and leftward errors are respectively defined as positive and negative, but the reference can also vary without altering the qualitative predictions. The blue line represents the standard risk-neutral model where α = β = 1. As expected, a proportional relationship between adaptation on the following trial and error on the previous trial is observed. However, when rightward, positive errors are penalized more than leftward, negative errors (α = 1.2, β = 0.8), adaptation is more sensitive to rightward than leftward errors. Another way to quantify this difference, which is better suited to the present experiment, is to compare adaptation to the risk-neutral case. Adaptation to large leftward errors is less sensitive than in the risk-neutral condition, whereas adaptation to large rightward errors is more sensitive (Figure 3, left panel). Also shown in Figure 3 are model predictions for when *only* leftward negative errors are penalized less, compared to the risk-neutral case, and when *only* rightward positive errors are penalized more than the risk-neutral case. They are depicted as the dashed red lines in the right top and bottom panels, respectively.

In the present experiment, our independent variable is subjective value, which is modulated by the experimental environment: Stable vs. Unstable. Therefore, we compared adaptation to identical error magnitudes *between* environments. Based on these model predictions we expected that, in the Unstable environment, participants would increase their sensitivity, adapting more to rightward errors. Additionally, or alternatively, participants may reduce their sensitivity, adapting less to leftward errors, compared to the Stable environment. This non-intuitive prediction emerges from the leftward direction of the force field and rightward location of the instability. To adapt to the leftward forces, they must push to the right. However, since the magnitude of the perturbation changes from trial to trial, overcompensating and adapting too strongly could lead to an undesirable rightward error beyond the cliff boundary. We expected that the more costly consequences of a rightward error in the Unstable environment would lead to weaker, less sensitive adaptation to leftward errors and/or stronger, more sensitive adaptation to rightward errors.

### Participants

Twenty healthy right-handed participants were recruited for the study. Nine participants performed the main experiment (mean ± *SD*: 22.3 ± 3.6 years, three females). Six participants performed the reverse experiment (23.33 ± 2.0 years, 4 males), and the remaining five participants performed the control experiment (21.0 ± 1.9 years, 5 males). Handedness was assessed with the Edinburgh Handedness Inventory (Oldfield, 1971). All participants provided informed consent and all methods were approved by the institutional review board at the University of Colorado, Boulder.

### Task

The task involved making horizontal reaching movements while seated and grasping the handle of a robotic arm (Interactive Motion Technologies, Shoulder-Elbow Planar Robot. The position of the handle was provided on each trial in the form of a yellow, circular cursor on a computer screen in front of the participants (Figure 1). Participants were asked to use the handle to steer the cursor from a start circle to a target circle 15 cm away in the sagittal plane. Start, target, and cursor circles had diameters of 0.70, 0.85, and 0.45 cm, respectively. Participants were secured in the seat with a four-point seat belt such that the trunk was restrained. Visual feedback of the cursor position was provided on a computer screen located above the robotic arm, directly in front of them at eye-level. They were instructed to make a rapid reaching movement to the target within a time window of 400–700 ms. Color-coded visual feedback of the reaching movements was provided about movement time. If participants reached the target within the correct time interval, the target would “blow up” (a yellow ring would emanate from the target). If they moved too fast or too slow, the target would turn green or gray, respectively. Participants were instructed to try and “blow up” the target on each trial.

### Protocol

The experimental protocol consisted of four phases: Baseline, Stable, Unstable, and Washout (Figure 2). Participants were not informed of the different phases. The Baseline phase consisted of 50 null trials (no-force) in which participants were allowed to familiarize themselves with the task and the time constraint. This was followed by the Stable phase, in which participants were exposed to a counter-clockwise viscous curl force field. In these force trials the robot handle exerted a force upon the participants' hands that had a magnitude proportional to handle velocity and acted in a direction perpendicular to the handle velocity. The robot-generated force was determined with the following equation:

(6) $$\left[\begin{array}{c} F_{x}\\ F_{y} \end{array}\right]=B\left[\begin{array}{cc} \;0 & 1\\ -1 & 0 \end{array}\right]\left[\begin{array}{c} V_{x}\\ V_{y} \end{array}\right]$$

In the above equation, *F*_*x*_ and *F*_*y*_ represent the handle force in the *x* and *y* directions, respectively. Handle velocity is represented by *V*_*x*_ and *V*_*y*_. The gain of the force field is represented by *B*. The gain of the force field for each trial was randomly selected, based on a uniform distribution, from 11 discrete bins equally spaced from 0 to −40 Ns/m. The Stable phase consisted of 200 trials, in which participants were exposed to each gain 18 times in random order. The Stable phase was followed by 400 trials in the Unstable phase. The sequence of gains in the Unstable phase was identical to the sequence of gains in the Stable phase. All participants were exposed to the same sequence. Just before trial 251, a white line appeared 2.5 cm to the right of the start circle and the target circle (Figure 1). The force field (6) was maintained to the left of the white line in the Unstable phase. However, if the handle moved more than 2.5 cm to the right of the start circle (beyond the white line), the robot generated cliff-like dynamics on the handle. This virtual cliff was presented as a divergent force field (Equation 7, *d* = 500 N/m), which resulted in a force that pulled the hand strongly to the right with a force proportional to the handle's horizontal, *x*, distance from the start circle:

(7) $$\left[\begin{array}{c} F_{x}\\ F_{y} \end{array}\right]=d\left[\begin{array}{cc} 1 & 0\\ 0 & 0 \end{array}\right]\left[\begin{array}{c} x\\ y \end{array}\right]$$

The divergent force field was present only in the region to the right of the white line, and was accompanied by an audiovisual cue that the participants had crossed the line (a bright red screen with black text indicating that the participants had crossed the “cliff”). While no explicit instructions were given regarding the presence of the white line, participants inevitably crossed the line, which resulted in experiencing the cliff-like dynamics. The divergent field was only present for the initial 50 trials of the Unstable phase. After the initial 50 trials the divergent field was removed, leaving only the audiovisual cue to indicate that the participants had crossed the line. For the remaining trials in the Unstable phase, participants were not adapting in the presence of a true instability, but rather the threat of instability. Importantly, the distance to the cliff was specifically chosen to be greater than the majority of movement errors, such that participants rarely crossed the cliff but were merely alerted to its presence. The Unstable phase was followed by the Washout phase, which consisted of 50 null (no force) trials to washout adaptation to the novel dynamics.

### Rationale for the design of the unstable phase

When participants are exposed to environments that are unstable throughout the workspace, they adapt by increasing joint stiffness through muscle coactivation (Burdet et al., 2001; Franklin et al., 2003; Selen et al., 2009). This suggests that participants could adapt to the instability presented in this experiment by using such a stiffening strategy, rather than adapting their feedforward control from trial to trial. To avoid this we created an environment that was unstable only in one region of the workspace. However, if participants still increased joint stiffness to compensate for the dynamics, this would manifest as a change in our experimental measure of feedforward adaptation (Franklin et al., 2003). Therefore, we created an environment with asymmetric instability to disambiguate any changes in feedforward adaptation from changes in stiffness control. Changes in stiffness control would lead to symmetric changes in adaptation, whereas changes in feedforward adaptation to a given gain would lead to asymmetric adaptation. A number of other precautions were taken to ensure that participants minimized the use of a stiffness strategy to counteract the instability. We placed the cliff boundary beyond the limit of normally experienced movement errors so that participants could theoretically use the same movement trajectories, the same stiffness, and the same adaptation pattern used in the Stable environment to successfully perform the task in the Unstable environment. The strength of the instability was also set so that it was very unlikely that participants could recover even if they did use a stiffness strategy. Finally, to rule out the effect of the unstable dynamics on the movement and adaptation, the divergent force field was removed after the first 50 trials. The unstable dynamics, if experienced, would change the movement kinematics and make it impossible to distinguish between changes due to changes in adaptation and changes simply due to the mechanical perturbation experienced. Removing the forces after 50 trials, without informing the participant, allowed us to induce the threat of instability without having unstable forces influence the adaptation metrics.

### Data acquisition and analysis

**Figure 4:**
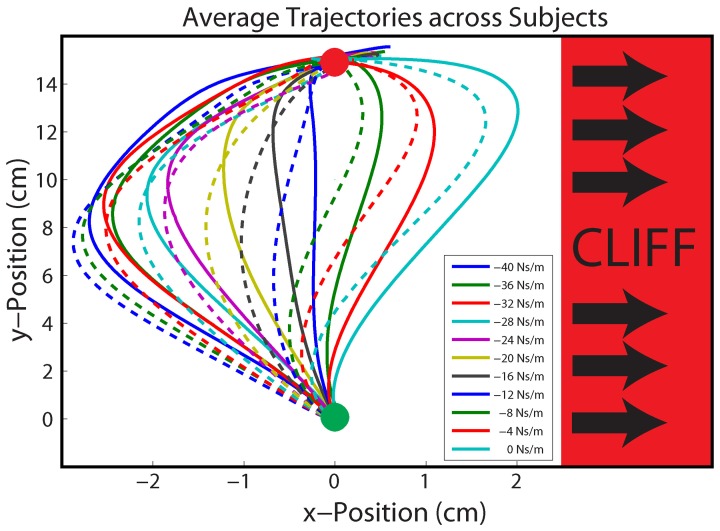
**Average Hand Paths**. Average hand paths across all participants to identical gains in the Stable (solid lines) and Unstable (dashed lines) phases. Participants reached from the start (green) circle to the target (red) circle. Movement errors were sampled 5 cm into the movement (horizontal black line). The unstable cliff region is depicted in red, right of center.

Robot handle position, handle velocity, and robot generated force were recorded at 200 Hz. Movement error was calculated as the perpendicular displacement from a line connecting the start and target circles. Movement error was measured early in the reaching movement (5 cm in the y-direction from the center of the home circle, Figure 4), in an effort to capture the error due to feedforward control based on the previous trial, and not the reactive control employed to counter the perturbation on that trial. To quantify the average movement error experienced in response to each gain, the error from each trial was grouped with trials of the same gain during each phase.

### Adaptation analysis

To test our predictions we analyzed adaptation in three different ways, and compared adaptation between phases. Although our predictions are based on the relationship between movement error and adaptation, it is difficult to control for movement error experimentally. Instead, we controlled for trial gain, as a proxy for movement error, and thereby ensured an equal number of trials for each gain. It is not unreasonable to do so, as previous studies have shown a linear relationship between gain and error (Fine and Thoroughman, 2007). Thus, for our first adaptation metric we quantified the relationship between our behavioral measure of adaptation and the *gain* on the preceding trial. We expected stronger gains to represent increasingly leftward errors and weaker gains to represent increasingly rightward errors. Our second adaptation metric was a model-based measure of adaptation, also calculated as a function of *gain* on the preceding trial. Finally, we also calculated an adaptation metric based on the relationship between our behavioral measure of adaptation and *movement error* on the preceding trial. The three adaptation analyses are described in detail below. For all metrics, we expected that adaptation to leftward errors (the stronger gains) would be reduced in the Unstable phase compared with the Stable phase. We also expected that adaptation to rightward errors (the weaker gains) would be greater in the Unstable phase compared with the Stable phase.

#### Behavioral gain-based adaptation

Adaptation was calculated based on the error observed in each trial, as a function of the gain experienced on the previous trial. Because the gain on each trial influenced the movement error on that trial, it was necessary to normalize errors before quantifying adaptation. Movement errors were normalized to the average error for the gain experienced on that trial. In order to quantify the influence of the gain on the current trial, *n*, on adaptation in the following trial, *n* + 1, we compared the normalized error between the trials before and after the current trial. Adaptation resulting from a given trial was defined by (8), where error_*n* + 1_ is the normalized error from the following trial, and error_*n* − 1_ is the normalized error from the previous trial.

(8) $${\text{Adaptation}}_{n}={\text{error}}_{n+1}-{\text{error}}_{n-1}$$

Adaptation to the gain, *k*, on trial *n* was calculated as the average adaptation observed in response to all trials with gain *k*. For the Stable phase, all 200 trials were included in the analysis. In the Unstable phase, adaptation was calculated as the average adaptation observed over the final 350 trials, removing the first 50 trials when the divergent force field was active. We did not analyze these initial 50 trials because we did not want to analyze any failed trials where the hand was pulled strongly to the right by the divergent field. Furthermore, we wanted to ensure that we analyzed data after the participants had been alerted to the presence of the cliff. All participants experienced the divergent field at least once during the 50 initial trials in the Unstable phase.

#### Model gain-based adaptation

We also sought to calculate adaptation in another manner, to confirm that the results were not dependent upon our specific definition of adaptation, provided in (8). To do so, we used the following state-space model (Fine and Thoroughman, 2007), based on the model introduced earlier, to fit the adaptation parameters:

(9) $$x_{n}=D(B_{n}-{\hat{B}}_{n})$$

(10) $${\hat{B}}_{n+1}=A{\hat{B}}_{n}+\vec{S}\cdot {\vec{p}}_{n}$$

The output of this model, *x*_*n*_, is the movement error on the current trial, *n*. This error was dependent on a scalar *D*, which represents arm compliance and the error in the prediction of the gain on the current trial (where *B*_*n*_ is the actual gain on the current trial, and ${\hat{B}}_{n}$ is the estimated gain for that trial). Equation 10 describes how the estimated gain for the following trial, ${\hat{B}}_{n+1}$, is dependent on the estimated gain on the current trial ${\hat{B}}_{n}$, a scalar *A*, which represents a forgetting factor as well as a sensitivity vector, $\vec{S}$. Each element of this vector corresponds to how much the gain experienced on that trial will contribute to the estimate on the following trial. For example, assuming a linear relationship between adaptation and error, if the error resulting from a given gain was large, then the corresponding term in the sensitivity vector should be large. If no error resulted from a given gain, then the corresponding term in the sensitivity vector should be zero. To ensure that only the element of the sensitivity vector corresponding to the specific gain experienced contributes to the estimation, the vector ${\vec{p}}_{n}$ is designed such that all elements of ${\vec{p}}_{n}$ are zero except the element corresponding to the gain experienced. The element corresponding to the gain experienced was set equal to 1. Using this definition, if adaptation is proportional to error, *K* from (1) is constant, then the sensitivity vector, $\vec{S}$, should scale linearly with gain and be identical across Stable and Unstable conditions. This definition is slightly different from other definitions of sensitivity in the literature, which would involve further normalization by the corresponding gain experienced to equate it to the constant *K* from (1). We fit the model by obtaining the *D*, *A*, and $\vec{S}$ that minimized the squared difference between the model predictions and the participant data in the final 200 trials of each phase using the built-in MATLAB function “fminsearch” (MATLAB). Our model-based measure of adaptation is the sensitivity vector. Its gain-specific elements are analogous to the gain-specific adaptation metric calculated from the behavioral data. The advantage of this analysis is that sensitivity does not depend on our experimentally derived calculation of adaptation, but only on the error experienced from trial-to-trial.

#### Behavioral error-based adaptation

Because our predictions are specifically based on adaptation to movement errors, a third analysis was performed on the experimental data, grouping the adaptation by experimental movement error rather than by trial gain. Trials were sorted into eleven 0.5 cm bins ranging from −3.0 cm through 2.5 cm. Leftward errors greater than 3.0 cm and rightward errors greater than 1.5 cm were excluded from the analysis. This size and range of the bins was selected to ensure an even distribution of trials between the bins, while minimizing the number of trials excluded from the analysis. Specifically, if there were an anomalously small number of trials in a given bin (<50), and any subject did not have a single trial in that bin, the bin was removed from the analysis.

### Reverse experiment

To further explore our hypotheses regarding subjective value, a second experiment was conducted in which the experimental setup was reversed. The cliff edge was placed to the left of the center of the screen, and the associated divergent force field pushed the handle to the left if the cursor was placed to the left of the cliff. The curl force field now pushed the handle to the right such that participants were still pushed away from the cliff edge as in the main experiment. Otherwise all experimental parameters remained the same, such as trial number, trial type, force magnitude and distribution. Six naive participants completed this reverse experiment and we compared adaptation between the Stable and Unstable conditions. In this experiment, because the placement of the cliff increased the subjective value of leftward errors relative to rightward errors, the predictions were reversed, although the hypotheses conceptually remained the same. Participants were expected to reduce adaptation to rightward errors (away from the cliff) and/or increase adaptation to leftward errors (toward the cliff).

### Control experiment

To explore the possibility that participants might reduce adaptation as a result of repeated exposure to the distribution of gains, a control experiment was conducted. Naive participants performed identical reaching movements as in the main experiment, with an identical set of gains, but without the presence of the unstable cliff. In this experiment, the white line that represented the edge of the cliff never appeared, nor did the divergent forces that were present beyond the cliff edge in the Main and Reverse experiment. The experiment consisted of four phases: Baseline, Early Stable, Late Stable, and Washout. The Baseline and Washout phases were identical to those in the main experiment, while the Early and Late Stable phase in the Control experiment consisted of a total of 600 reaching trials dynamically identical to those in the Stable Phase of main experiment. Since there was no Unstable phase in this experiment, we renamed the phases for clarity. The Early Stable and Late Stable phases correspond, in terms of trial number, to the Stable and Unstable phases of the main experiment: the first 200 force field trials and the last 350 force field trials, respectively.

### Statistics

In this study we altered the subjective value of error between the Stable and Unstable phases. To determine whether subjective value of error influenced adaptation, we used a linear mixed effects regression model to analyze the error and adaptation observed in each experiment. The mixed effects model was selected because of its ability to consider changes between the phases of the experiment while considering intra-participant variability. It is called a “mixed” effects model because it models both random and fixed effects. In this case, the within subject (random) and between subject (fixed) effects are both taken into account in the final output of the model. First, we determined whether gain was a suitable proxy for error by including gain as a factor. We also included phase as a factor to test whether there was a difference in error between the two phases. A phase by gain interaction term was also included to determine whether there were differential effects of phase at individual gains. To confirm that adaptation was influenced by gain we included gain in the model as a factor. We also included phase as a factor to determine whether phase influenced adaptation. Finally, to determine whether adaptation to the larger gains (leftward errors) and/or smaller gains (rightward errors) were differentially affected by phase, we also included a phase by gain interaction term. Planned comparisons were carried out on adaptation between phases to the strongest and weakest gains or to the most leftward and rightward errors (for the error-based adaptation). For the model gain-based adaptation, sensitivity, rather than gain, was included as a factor as well as a sensitivity by phase interaction term. Similarly, for the error-based behavioral adaptation, movement error, rather than gain, was included as a factor as well as an error by phase interaction term. The level for statistical significance was set at α = 0.05.

## Results

### Overview

In order to determine whether the subjective value of an error can modulate adaptation we quantified adaptation to random perturbations during reaching movements in two novel dynamic environments: (1) a stable environment, and (2) an unstable environment in which we altered the subjective value of rightward movement errors compared with leftward errors (Figure 2). Overall we found that on average, movement error was different between the two environments and that the subjective value of movement error of a given magnitude significantly affected adaptation. The behavioral results of this study are supported by model-derived changes in sensitivity to gain when the subjective value of an error was altered.

### Movement error

**Figure 5:**
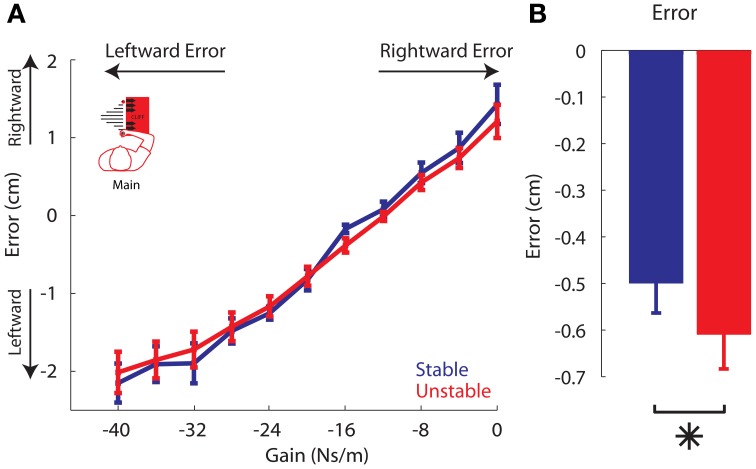
**Movement error vs. Gain. (A)** Average movement errors across all participants, for each trial gain, are plotted for the Stable (blue) and Unstable (red) phases. **(B)** Average error in each phase (positive: rightward, negative: leftward). The Unstable phase had slightly greater leftward error. Asterisk indicates *P* < 0.05. Error bars represent standard error of the mean.

We began our analysis by examining the movement error for each gain. In both phases, rightward errors greater than 2.5 cm were rare. Trial movement error was grouped by gain into bins and separated by phase. The results of the linear mixed effects regression model indicated that there was a main effect of gain (*P* < 0.00001), and confirmed a linear relationship between movement error and gain in each phase. Stronger gains led to increasingly leftward errors, and weaker gains led to increasingly rightward errors (Figure 5A). These results support the use of gain as a proxy for error in adaptation analyses to come.

If adaptation differed between the two phases, this should influence the average movement trajectories and, accordingly, the average movement error. Specifically, if adaptation to leftward errors was reduced and adaptation to rightward error increased in the Unstable phase, then movement errors should be more leftward, away from the cliff. Indeed, the linear mixed effects regression model also indicated there was a main effect of phase. In other words, there was a significant difference in movement error between phases (*P* = 0.0154; Figure 5B); there were more leftward trajectories, and leftward errors in the Unstable compared with the Stable phase (Figures 4, 5B). The average movement error in the Stable phase was −0.5 ± 0.19 cm, slightly less leftward than the average movement error in the following Unstable phase −0.61 ± 0.22 cm. There was no phase × gain interaction (*P* = 0.0864).

Participants moved with similar velocities in both conditions (paired *t*-test, *P* = 0.7926), with average velocities of 43 ± 14 cm/s and 43 ± 15 cm/s in the Stable and Unstable phases, respectively. The similarity of hand velocities indicates that participants experienced similar velocity-dependent robot forces in both phases.

### Behavioral gain-based adaptation

**Figure 6:**
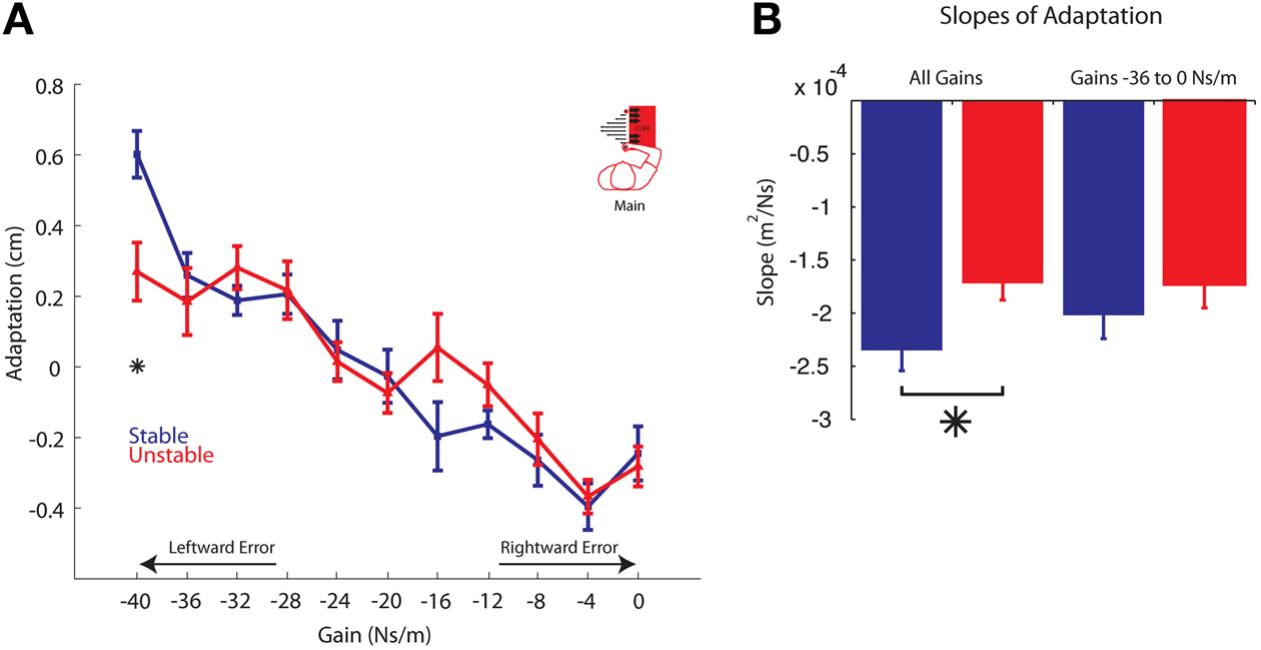
**Adaptation vs. Gain. (A)** Average adaptation for all participants vs. gain is plotted for the Stable (blue) and Unstable (red) phases. For clarity, arrows are used to indicate gains resulting in increasingly leftward or rightward errors. **(B)** Slopes of the adaptation curves for all gains (left) and only gains −36 through 0 Ns/m (right). Asterisks indicate *P* < 0.05. Error bars represent standard error of the mean.

In the Stable phase, the average adaptation plotted as a function of the gain on the previous trial displays a linear relationship (Figure 6A). The slope of this line, fit using a linear mixed effects regression model, was found to be significant (*P* < 0.00001, *k* = −2.24 m^2^/Ns). There was also a significant effect of gain on adaptation in the Unstable phase (*P* < 0.00001; Figure 6A). The slope of the adaptation curve for the Unstable phase (*k* = −1.72 cm^2/Ns)^ was significantly different from that of the adaptation curve in the Stable phase (*P* = 0.0043; Figure 6B), indicating a significant effect of phase. The cliff by gain interaction term was also significant (*P* = 0.0306), indicating that the presence of instability led to greater differences in adaptation at some gains more than others. Indeed, the adaptation vs. gain relationship for the Unstable phase (Figure 6A) qualitatively displays a notable non-linearity at the strongest gain, but otherwise is similar to the adaptation observed in the Stable phase. To further quantify this difference, planned comparisons were performed and revealed a significant reduction in adaptation to the strongest gain (*P* = 0.005), and similar adaptation to all other gains (*P* > 0.05). This is in line with the model predictions presented in Figure 3. Specifically, similar results are prediction by a model exhibiting reduced adaptation to leftward errors only as the strongest gain led to the greatest leftward errors. We chose to quantify adaptation over the final 350 trials in the Unstable phase to maximize the data included in the analysis. Nevertheless, the changes observed between phases appear to be long-lasting; there was no difference in adaptation between the final 200 trials in the Unstable phase compared to the final 350 trials (*P* = 0.600, linear mixed effects regression).

Because the relationship between adaptation and gain appears to exhibit a non-linearity to the strongest gain (−40 Ns/m), we also explored the possibility that the reduction in adaptation to the strongest gain was significant enough to alone cause the change in the slopes of these curves. An identical analysis was performed on the data set after removing those data associated with the strongest gain. When we excluded the data corresponding to adaptation at the strongest gain, there was no longer a significant difference in the slopes of the adaptation curves for each phase (*P* = 0.315; Figure 6B).

### Model gain-based adaptation

**Figure 7:**
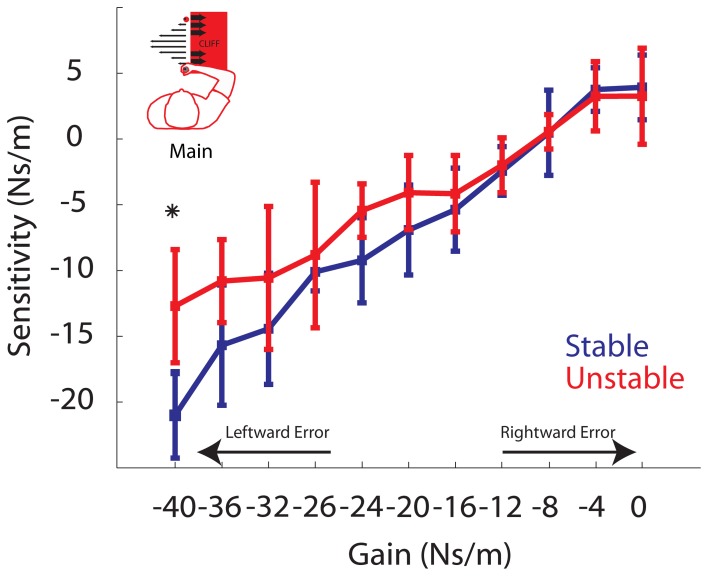
**Sensitivity vs. Gain**. Group averaged elements of the sensitivity vector are plotted for Stable (blue) and Unstable (red) phases. For clarity, arrows are used to indicate gains resulting in increasingly leftward or rightward errors. Asterisks indicate *P* < 0.05. Error bars represent standard error of the mean.

We next turn to the model gain-based analysis of adaptation. While the elements of the sensitivity vector, *S*, varied from participant to participant, the values of *D* (2.2e-4 ± 0.46e-4 m^2^/Ns) and *A* (0.69 ± 0.08) are consistent with previous findings (Fine and Thoroughman, 2007). Model fits predicted the data well with an average *R*^2^ of 0.76 ± 0.002 across participants. The elements of the sensitivity vector exhibit a linear positive relationship with gain in both phases (Figure 7). Again these data were analyzed using the linear mixed effects regression model with gain and phase included as factors, as well as a gain by phase interaction term. The slope of the sensitivity vs. gain curve during both the Stable and Unstable phase were found to be significant: *P* < 0.002, *k* = 0.60519 and *P* < 0.002, *k* = 0.4188, respectively. As in the behavioral results, there was a significant gain by phase interaction (*P* = 0.0000168), a planned comparison at the largest gain indicates a significant difference (*P* = 0.0006; *B* = −40 Ns/m). Specifically, sensitivity to this gain was reduced in the Unstable phase compared to the Stable phase. *Post-hoc* pairwise comparisons also reveal a trend toward a reduction in sensitivity to gains *B* = −36 Ns/m, *B* = −24 Ns/m, and −20 Ns/m (*P* = 0.0222, *P* = 0.0107, *P* = 0.0435). However, when correcting for multiple comparisons these *P*-values are greater than the Bonferroni-corrected significance level (α = 0.05/9 = 0.0056). Overall, these model results confirm our behavioral findings. They indicate that sensitivity, the model-based metric of adaptation, was also affected by phase, and that sensitivity to the larger gains was reduced in the Unstable phase compared to the Stable phase. These findings are also in line with the predictions in Figure 3 in the case of reduced adaptation to leftward errors only (i.e., the strongest gains). Moreover, compared to the behavioral results, the sensitivity analysis reveals a stronger effect of phase on sensitivity. We observed reduced sensitivity in four of the highest gains, compared to the observation of reduced adaptation to only the largest gain in the behavioral analysis.

### Behavioral error-based adaptation

**Figure 8:**
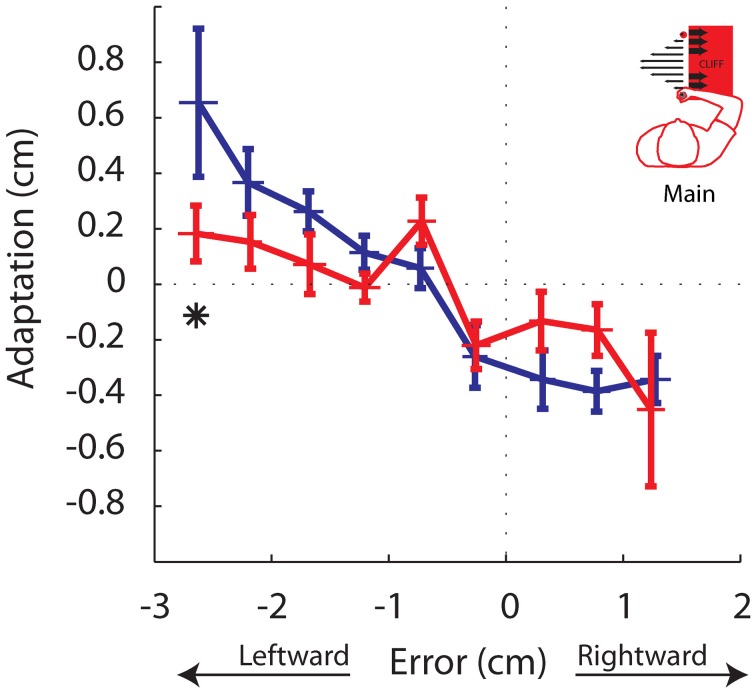
**Adaptation vs. Movement Error**. Adaptation for each trial was binned by the magnitude of the error experienced on that trial. Each data point represents the the average adaptation and the average error across participants for a given error bin, for the Stable (blue) and Unstable (red) phases. Asterisks indicate *P* < 0.05. Error bars indicate standard error of the mean.

Finally, we performed an error-based analysis. Although the experimental design did not explicitly control for error, this analysis would provide confirmation, albeit inherently variable, that adaptation to a given error differed between phases. Despite the increased variability in the results, the analysis supported the findings of the gain-based analysis. Trials were sorted by the magnitude of movement error into 11 bins, each 0.5 cm wide, ranging from −3.0 cm through 1.5 cm. The same bins were used for all participants. Similar to the gain-based analysis, effect of bin and phase were observed, as well as a bin by phase interaction (*P* < 0.00005, *P* = 0.0363, and *P* = 0.0322, respectively). In the Unstable phase participants significantly reduced adaptation in response to the largest leftward movement errors (*P* = 0.0471; Figure 8). These findings are also in line with the predictions in Figure 3 in the case of reduced adaptation to leftward errors only. It is interesting that participants also slightly reduced adaptation in response to small rightward movement errors between 0.5 and 1.0 cm, although this was not significant when corrected for multiple comparisons (*P* = 0.0483; Figure 8). Thus, this error-based adaptation reveals weaker adaptation to leftward errors and also suggests that the reference line that defined whether an error was non-threatening (away from the cliff) or threatening (toward the cliff), may not have been strictly defined by a straight line toward between the home and the target. It is reasonable that participants may have considered small rightward errors as non-threatening. A slight rightward offset in the reference line could still lead to more leftward errors on average and trajectories that avoided the cliff, which is what was observed (Figure 5B). It would be interesting in future investigations to understand what factors drive this distinction.

### Reverse experiment

**Figure 9:**
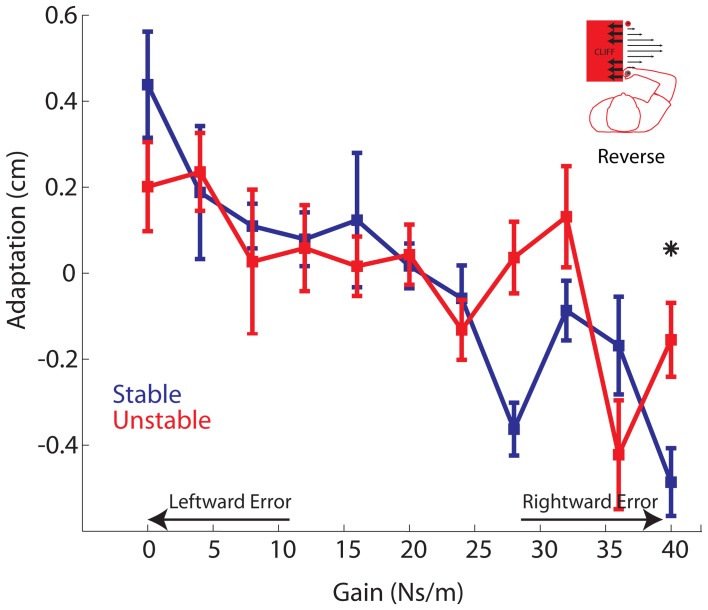
**Reverse Experiment**. Adaptation vs. Gain. Average adaptation for all participants vs. gain is plotted for the Stable (blue) and Unstable (red) phases. For clarity, arrows are used to indicate gains resulting in increasingly leftward or rightward errors. Asterisks indicate *P* < 0.05. Error bars represent standard error of the mean.

In the main experiment, the cliff was located to right, increasing the subjective value of rightward errors compared with leftward errors. In the Reverse experiment, we reflected the cliff location so that leftward errors had a greater subjective value than rightward errors. As in the main experiment, we compared both movement error and adaptation between the Stable and Unstable phases. As expected, there was a main effect of gain in both the error and adaptation analyses (both *P*'s < 0.00001). Similar to the main experiment, movement error differed significantly between Stable and Unstable phases (Stable: 0.89 ± 0.96 cm; Unstable: 1.05 ± 1.22 cm; *P* = 0.0209), Movement errors were more rightward in the Unstable phase indicative of a desire to avoid the cliff. Turning to the adaptation results, there was a significant phase by gain interaction (*P* = 0.0121). We observed that adaptation to the strongest rightward gains, leading to the largest rightward errors, was significantly reduced in the Unstable phase compared with the Stable phase (Figure 9). Specifically, a planned comparison revealed that adaptation to the strongest rightward gain of *B* = 40 Ns/m was significantly reduced in the Unstable compared with the Stable phase (*P* = 0.016). There was also a trend toward reduced adaptation to the gain of *B* = 28 Ns/m, however this was not significant when corrected for multiple comparisons (*P* = 0.011). Such reduced adaptation to the strongest rightward gain is in direct contrast to the findings of the main experiment where adaptation to the strongest leftward gains, leading to the largest leftward errors, was reduced. However, this reversal is precisely what we expected to occur given that the location of the cliff and subjective value of the movement error was also reversed.

### Control experiment

**Figure 10:**
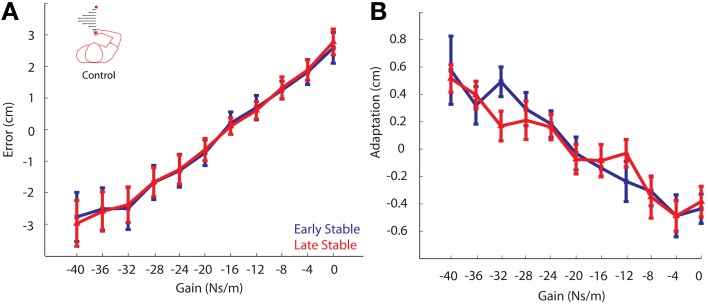
**Control Experiment. (A)** Movement error vs. Gain. Error from the Early Stable phase is shown in blue, while error from the Late Stable phase is shown in red. There was no difference in movement errors between phases. **(B)** Adaptation vs. Gain. Adaptation for all participants vs. gain for the Early Stable phase and Late Stable phase. No significant changes in adaptation were found to any gain. Error bars represent standard error of the mean.

To ensure that the changes in adaptation between the phases were not simply the result of prolonged exposure to the viscous curl field, a control experiment was conducted in which participants made 650 reaching movements without the presence of the unstable cliff region. Similar to the main experiment, we compared movement error and gain-based behavioral adaptation between phases. These data were also analyzed using the linear mixed effects regression model with gain and phase included as factors, and a gain by phase interaction term. As expected, there was a main effect of gain in both the error and adaptation analyses (both *P*'s < 0.00001). We found there was no difference in movement error between the Early Stable Phase and the Late Stable Phase (Early Stable: −0.43 ± 0.96; Late Stable: −0.44 ± 0.78 cm; *P* = 0.872; Figure 10A). Similarly, no difference in adaptation was found between the Early Stable and Late Stable Phases in the control experiment (*P* = 0.5576; Figure 10B). It is also important to note the consistency in the magnitude of adaptation observed in response the largest gain in the Control experiment (Figure 10B: gain = −40 Ns/m), where the environment is always Stable, and the adaptation to the largest gain observed in the Stable phase of the main experiment (Figure 6A: gain = −40 Ns/m). This strengthens our finding that adaptation to the largest gain was indeed reduced in the Unstable phase of the Main experiment.

### Summary

**Figure 11:**
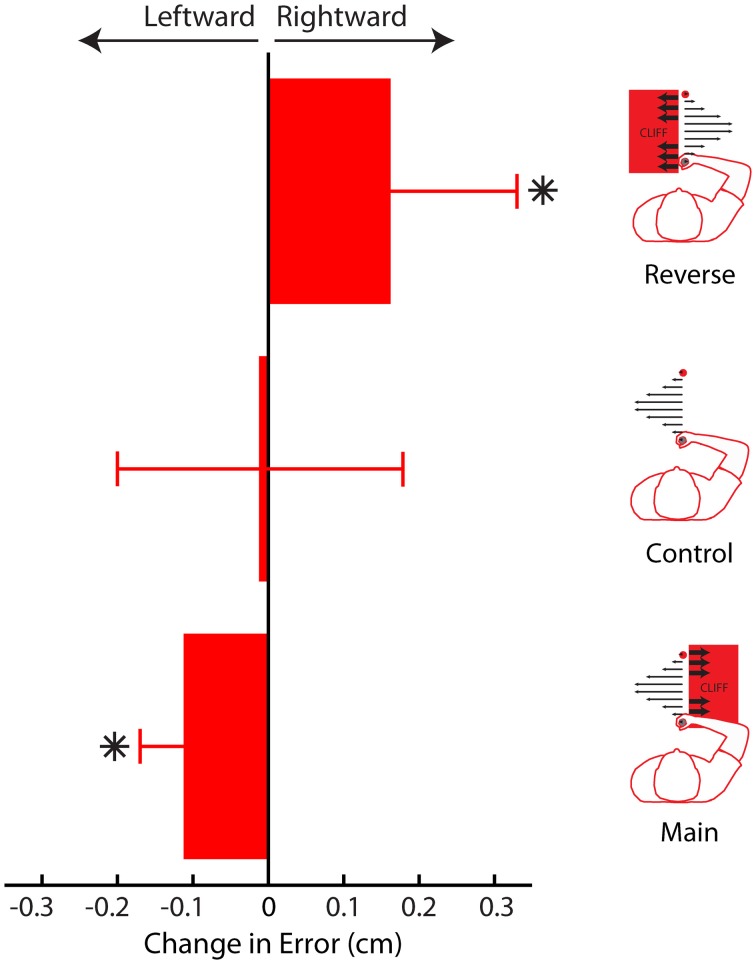
**Average change in movement error between phases for Main, Control, and Reverse experiments**. In both the Main and Reverse experiments, participants' movement trajectories avoided the cliff in the later phase (when the cliff was present). In the Control experiment, no change was detected. Error bars represent standard error of the mean. Asterisks indicate *P* < 0.05.

Here we have presented results from three different experiments, using three different analyses demonstrating that subjective value can influence adaptation, and ultimately adapted behavior. When the cliff was on the right, adaptation to leftward errors, away from the cliff, decreased, leading to greater leftward errors (away from the cliff, Figure 11). When the cliff was on the left, adaptation to rightward errors decreased, leading to greater rightward errors (away from the cliff). Finally, when no cliff was present, errors did not change, and no change in adaptation was detected.

## Discussion

In this study, we investigated the influence of the subjective value of movement error on adaptation during a novel reaching task. We found that introducing a cliff-like region in the workspace, and thereby changing the subjective value of error, we could modulate the degree to which participants would adapt to movement errors of the same magnitude. Weaker adaptation was observed in response to movement errors away from the cliff in the Unstable phase, when such errors had lower subjective value. These results are a demonstration of a risk-sensitive process in movement adaptation, in that adaptation was influenced by the subjective value of error rather than solely the magnitude of error. Our findings indicate that we don't simply learn from our mistakes, we may also learn from how much we value our mistakes.

It is intriguing that participants primarily demonstrated reduced adaptation to leftward errors away from the cliff (the strongest gains). They could have additionally, or alternatively, demonstrated stronger adaptation to the rightward errors toward the cliff (the weakest gains). However, all three analyses consistently demonstrated weaker adaptation to leftward errors. Even in the Reverse experiment, only weaker adaptation to rightward errors (away from the cliff) was observed. Why not use both strategies? First, let us emphasize that both strategies lead to overall under-compensation for the force field, and increasing errors away from the cliff. This is not surprising, as one would like to avoid the cliff as much as possible. However, the target must still be reached, and greater errors may compromise one's ability to reach the target within the time constraints. Participants may have realized that weaker adaptation to the leftward gains, under-compensating for only the strongest gains, sufficiently allowed them to avoid the cliff, yet reduce leftward errors as well. Under-compensation for all the gains would have reduced the possibility of crossing the cliff boundary even more, but at the expense of increased leftward errors. By reducing adaptation and under-compensating only to the largest gains, participants were effectively optimizing a tradeoff between performing the task and avoiding the worst-case scenario.

A critical element of the experimental design is that participants changed how they adapted without regularly experiencing the penalty. Because the forces experienced in the Stable phase caused movement errors away from the cliff region, participants rarely experienced errors large enough in magnitude to result in the cursor entering the unstable region. It was merely the threat of instability that led to changes in adaptation, not the instability itself or the surprise associated with the initial experience with the instability. The instability was actually not present for most of the experiment. These results demonstrate that adaptation can be modulated indirectly without explicitly constraining movement.

We propose our observation of a difference in adaptation is evidence of risk-sensitivity in the learning process, where risk-sensitivity is defined as sensitivity to the variance over outcomes (i.e., error). Although we do not explicitly modulate the error variance in this task, a distortion between the subjective value and actual value of an error will manifest as risk-sensitive behavior. Thus, a strong prediction that emerges from these results is that increasing or decreasing the variance of error in an environment that resembles the cliff-like environment created in the present study, will alter the adaptation process.

### Relationship to error-based learning studies

Recent studies have shown that subtle changes in the properties of a given movement error can drive distinct changes in properties of the adaptation process (such as savings and generalization to other movement contexts; Kluzik et al., 2008; Berniker and Kording, 2011). However, in all these studies the magnitude of error also varied. We report significant changes in adaptation in response to movement errors of the same magnitude, clearly demonstrating that adaptation does not depend solely on error magnitude. Changing the subjective value of error by penalizing some errors more than others can modulate adaptation.

### Relationship to reward-based learning studies

The subjective value of an error could be interpreted as the reward associated with an error. Surprisingly, only a few studies have examined the influence of reward, or the role of reinforcement learning, on movement adaptation. One such study demonstrated that participants could learn a movement task using only reward feedback, in the absence of sensory feedback of the error (Izawa and Shadmehr, 2011). The authors concluded that reward feedback may harness reward learning mechanisms over and above the error-driven learning. This may be relevant for our findings as well. Changing the subjective value of error may recruit striatal mechanisms in the adaptation process, in addition to the well-documented cerebellar mechanisms (Smith and Shadmehr, 2005; Rabe et al., 2009; Bastian, 2011).

While risk-sensitivity has been assessed in single movements (Wu et al., 2009; Braun et al., 2011; O'Brien and Ahmed, 2013), as well as sequences of decisions (Averbeck et al., 2011; Niv et al., 2012), to our knowledge this is the first demonstration of risk-sensitivity in sequences of movement decisions (i.e., movement adaptation). Niv et al. demonstrated non-linear learning in sequences of non-motor decisions and concluded that the non-linearity is associated with a risk-sensitive learning process that penalizes positive and negative errors asymmetrically. In the present study we also observe that large leftward errors (a consequence of the largest gains) lead to weaker adaptation than large rightward errors (a consequence of the weakest gains). However, while risk-sensitivity in the study by Niv and colleagues appears to emerge from an inherent distortion between positive and negative errors, we explicitly penalize rightward errors more than leftward errors in our experimental task.

In the present study, subjective value was modulated by increasing the penalty associated with a given movement error. In other words, we provided negative rewards, and cannot necessarily extrapolate our findings to conditions where subjective value is altered by modulating positive rewards. A mounting body of evidence over the past few years has indicated that positive and negative reward differentially affect decision making. Results of a recent study seem to indicate that participants relied more heavily on positive feedback (Averbeck et al., 2011). Even in skill learning, positive reward leads to improved retention over days to months. While our study only modulated the degree of negative reward, we nevertheless observed that it could influence adaptation. An interesting question for future research is the whether modulating positive reward would have led to more pronounced differences in movement adaptation.

It may be argued that the risk-sensitive behavior demonstrated in this task emerges only because we have explicitly designed it to, and does not represent natural movement tasks. In other words, humans do not inherently penalize rightward errors more than leftward errors; we explicitly designed a task that did just that. How then is this relevant to motor control? Such a distortion between the actual and subjective value of a movement error is inherent to many activities of daily living. The simple reaching movements frequently studied in laboratories over the past couple decades do not normally demonstrate this distortion so this phenomenon has been largely overlooked. But postural movements, which are inherently unstable and constrained within a given base of support, are a natural example of a situation where the magnitude of an error does not correspond to the subjective value of that error. A 2 cm movement error within the base of support, is very different that the same 2 cm error that moves the center of mass beyond the base of support. The latter will result in a loss of balance, the former will not. Results from a recent study suggest that postural learning is modulated asymmetrically by stability limits, suggesting that adaptation may be risk-sensitive (Manista and Ahmed, 2012). Indeed, we propose that motor adaptation in any environment where the error and its consequence do not correspond, will be risk-sensitive.

### Clinical relevance

The finding that adaptation can be modulated by changing subjective value is of great relevance to current rehabilitation programs for patients suffering from neurological impairments such as a stroke or Parkinson's disease. It may be possible to influence the adaptation process, in a manner tailored to each patient, by simply rewarding some movements more than others. Future studies should investigate alternative means of modulating subjective value, via implicit rewards like verbal instruction, encouragement or visual feedback. Alternatively, explicit rewards such as point rewards and penalty and/or monetary compensation could be investigated.

## Limitations

The experimental design prevented us from investigating participant's initial adaptation to the Unstable phase. It would be of interest to know how their adaptation changed during those initial 50 trials, but because of the variability and the small sample size during this period we were unable to perform this analysis. A second limitation is that the removal of the unstable forces after the initial 50 trials in the Unstable phase, may have influenced the strength of the overall effect. Participants did occasionally cross the cliff edge after the unstable forces were no longer present. While there was still an audiovisual penalty, the lack of unstable forces may have influenced the participants' avoidance of the cliff edge.

## Conclusions

In summary, our results provide evidence for a risk-sensitive process underlying movement adaptation. Adaptation can be altered by modulating an individual's subjective error value function. The implications of these findings are far-reaching and could potentially lead to new and improved rehabilitation therapies that are tailored to each and every patient at an individual level. More generally, we hope they can lead to significant advances in our understanding of the neural mechanisms by which risk influences the learning process in both motor and non-motor tasks.

## Author contributions

Michael C. Trent designed and performed research, analyzed data and wrote the paper. Alaa A. Ahmed designed and performed research, analyzed data, and wrote the paper.

### Conflict of interest statement

The authors declare that the research was conducted in the absence of any commercial or financial relationships that could be construed as a potential conflict of interest.

## References

[B1] Averbeck B. Kilner J. Frith C. (2011). Neural correlates of sequence learning with stochastic feedback. J. Cogn. Neurosci. 23, 1346–1357 10.1162/jocn.2010.21436 20146602 PMC3267427

[B2] Bastian A. (2011). Moving, sensing and learning with cerebellar damage. Curr. Opin. Neurobiol. 21, 596–601 10.1016/j.conb.2011.06.007 21733673 PMC3177958

[B3] Bastian A. J. (2008). Understanding sensorimotor adaptation and learning for rehabilitation. Curr. Opin. Neurol. 21, 628–633 10.1097/WCO.0b013e328315a293 18989103 PMC2954436

[B4] Berniker M. Kording K. (2011). Estimating the relevance of world disturbances to explain savings, interference and long-term motor adaptation effects. Plos Comput. Biol. 7:e1002210 10.1371/journal.pcbi.1002210 21998574 PMC3188508

[B5] Bernoulli D. (1954). Exposition of a new theory on the measurement of risk. Econometrica 22, 23–36 10.2307/1909829

[B6] Braun D. Nagengast A. Wolpert D. (2011). Risk-sensitivity in sensorimotor control. Front. Hum. Neurosci. 5:1 10.3389/fnhum.2011.00001 21283556 PMC3028548

[B7] Burdet E. Osu R. Franklin D. Milner T. Kawato M. (2001). The central nervous system stabilizes unstable dynamics by learning optimal impedance. Nature 414, 446–449 10.1038/35106566 11719805

[B8] Fine M. Thoroughman K. (2007). Trial-by-trial transformation of error into sensorimotor adaptation changes with environmental dynamics. J. Neurophysiol. 98, 1392–1404 10.1152/jn.00196.2007 17615136

[B9] Franklin D. Osu R. Burdet E. Kawato M. Milner T. (2003). Adaptation to stable and unstable dynamics achieved by combined impedance control and inverse dynamics model. J. Neurophysiol. 90, 3270–3282 10.1152/jn.01112.2002 14615432

[B10] Glimcher P. (2008). Understanding risk: a guide for the perplexed. Cogn. Affect. Behav. Neurosci. 8, 348–354 10.3758/CABN.8.4.348 19033233

[B11] Huettel S. Stowe C. Gordon E. Warner B. Platt M. (2006). Neural signatures of economic preferences for risk and ambiguity. Neuron 49, 765–775 10.1016/j.neuron.2006.01.024 16504951

[B12] Izawa J. Shadmehr R. (2011). Learning from sensory and reward prediction errors during motor adaptation. Plos Comput. Biol. 7:e1002012 10.1371/journal.pcbi.1002012 21423711 PMC3053313

[B13] Kahneman D. Tversky A. (1979). Prospect theory - analysis of decision under risk. Econometrica 47, 263–291 10.2307/1914185

[B14] Kluzik J. Diedrichsen J. Shadmehr R. Bastian A. (2008). Reach adaptation: what determines whether we learn an internal model of the tool or adapt the model of our arm. J. Neurophysiol. 100, 1455–1464 10.1152/jn.90334.2008 18596187 PMC2544452

[B15] Lackner J. Dizio P. (1994). Rapid adaptation to coriolis-force perturbations of arm trajectory. J. Neurophysiol. 72, 299–313 7965013 10.1152/jn.1994.72.1.299

[B16] Manista G. C. Ahmed A. A. (2012). Stability limits modulate whole-body motor learning. J. Neurophysiol. 107, 1952–1961 10.1152/jn.00983.2010 22236715

[B17] Marko M. K. Haith A. M. Harran M. D. Shadmehr R. (2012). Sensitivity to prediction error in reach adaptation. J. Neurophysiol. 108, 1752–1763 10.1152/jn.00177.2012 22773782 PMC3774589

[B18] Niv Y. Edlund J. Dayan P. O'Doherty J. (2012). Neural prediction errors reveal a risk-sensitive reinforcement-learning process in the human brain. J. Neurosci. 32, 551–562 10.1523/JNEUROSCI.5498-10.2012 22238090 PMC6621075

[B19] O'Brien M. K. Ahmed A. A. (2013). Does risk-sensitivity transfer across movements. J. Neurophysiol. 109, 1866–1875 10.1152/jn.00826.2012 23324319 PMC4073925

[B20] Oldfield R. (1971). The assessment and analysis of handednesss: the edinburgh inventory. Neuropsychologia 9, 97–113 10.1016/0028-3932(71)90067-4 5146491

[B21] Rabe K. Livne O. Gizewski E. Aurich V. Beck A. Timmann D. (2009). Adaptation to visuomotor rotation and force field perturbation is correlated to different brain areas in patients with cerebellar degeneration. J. Neurophysiol. 101, 1961–1971 10.1152/jn.91069.2008 19176608

[B22] Scheidt R. Dingwell J. Mussa-Ivaldi F. (2001). Learning to move amid uncertainty. J. Neurophysiol. 86, 971–985 11495965 10.1152/jn.2001.86.2.971

[B23] Selen L. Franklin D. Wolpert D. (2009). Impedance control reduces instability that arises from motor noise. J. Neurosci. 29, 12606–12616 10.1523/JNEUROSCI.2826-09.2009 19812335 PMC2784227

[B24] Shadmehr R. Mussa-Ivaldi F. (1994). Adaptive representation of dynamics during learning of a motor task. J. Neurosci. 14, 3208–3224 8182467 10.1523/JNEUROSCI.14-05-03208.1994 PMC6577492

[B25] Smith K. Dickhaut J. McCabe K. Pardo J. (2002). Neuronal substrates for choice under ambiguity, risk, gains, and losses. Manag. Sci. 48, 711–718 10.1287/mnsc.48.6.711.194

[B26] Smith M. Shadmehr R. (2005). Intact ability to learn internal models of arm dynamics in Huntington's disease but not cerebellar degeneration. J. Neurophysiol. 93, 2809–2821 10.1152/jn.00943.2004 15625094

[B27] Thoroughman K. Shadmehr R. (2000). Learning of action through adaptive combination of motor primitives. Nature 407, 742–747 10.1038/35037588 11048720 PMC2556237

[B28] Topka H. Massaquoi S. Benda N. Hallett M. (1998). Motor skill learning in patients with cerebellar degeneration. J. Neurol. Sci. 158, 164–172 10.1016/S0022-510X(98)00115-4 9702687

[B29] Wei K. Koerding K. (2009). Relevance of error: what drives motor adaptation. J. Neurophysiol. 101, 655–664 10.1152/jn.90545.2008 19019979 PMC2657056

[B30] Wu S. Delgado M. Maloney L. (2009). Economic decision-making compared with an equivalent motor task. Proc. Natl. Acad. Sci. U.S. A. 106, 6088–6093 10.1073/pnas.0900102106 19332799 PMC2669401

